# Functional Characterization of the *1-Deoxy-D-Xylulose 5-Phosphate Synthase Genes* in *Morus notabilis*

**DOI:** 10.3389/fpls.2020.01142

**Published:** 2020-07-24

**Authors:** Shaoyu Zhang, Guangyu Ding, Wenmin He, Kai Liu, Yiwei Luo, Jiaqi Tang, Ningjia He

**Affiliations:** ^1^ State Key Laboratory of Silkworm Genome Biology, Southwest University, Chongqing, China; ^2^ Industrial Engineering Research Center of Mulberry, State Forestry and Grassland Administration, Chongqing, China

**Keywords:** terpenoids, 1-deoxy-D-xylulose 5-phosphate synthase, *Morus notabilis*, functional differentiation, early flowering

## Abstract

Terpenoids are considered to be the largest group of secondary metabolites and natural products. Studies have revealed 1-deoxy-D-xylulose 5-phosphate synthase (DXS) is the first and rate-limiting enzyme in the plastidial methylerythritol phosphate pathway, which produces isopentenyl diphosphate and its isoform dimethylallyl diphosphate as terpenoid biosynthesis precursors. Mulberry (*Morus* L.) is an economically and ecologically important perennial tree with diverse secondary metabolites, including terpenoids that protect plants against bacteria and insects and may be useful for treating human diseases. However, there has been relatively little research regarding *DXS* genes in mulberry and other woody plant species. In this study, we cloned and functionally characterized three *Morus notabilis DXS* genes (*MnDXS1*, *MnDXS2A*, and *MnDXS2B*). Bioinformatics analyses indicated *MnDXS1* belongs to clade 1, whereas *MnDXS2A* and *MnDXS2B* are in clade 2. The three encoded MnDXS proteins are localized to chloroplasts. Additionally, substantial differences in *MnDXS* expression patterns were observed in diverse tissues and in response to insect feeding and methyl jasmonate treatment. Moreover, overexpression of *MnDXS1* in *Arabidopsis thaliana* increased the gibberellic acid content and resulted in early flowering, whereas overexpression of *MnDXS2A* enhanced root growth and increased the chlorophyll and carotenoid content. Our findings indicate that *MnDXS* functions vary among the clades, which may be useful for further elucidation of the functions of the *DXS* genes in mulberry.

## Introduction

Terpenoids, which are also known as terpenes, are the most diverse natural products that are widely distributed among various species, including higher plants, fungi, and bacteria. In plants, terpenoids are crucial for growth and development and have specialized functions related to responses to environmental stimuli (Zhang et al., 2019). Additionally, terpenoids have long been used in perfumes, pharmaceutical products, insecticides, and industrial compounds (Tholl, 2015). For example, artemisinin, which is a potent antimalarial drug, is a sesquiterpene endoperoxide isolated from *Artemisia annua* (Zheng et al., 2017; Zhang et al., 2018). The mechanisms underlying terpenoid biosynthesis need to be more comprehensively characterized to further elucidate terpenoid functions in plants and satisfy the demand for more sustainable terpenoid production methods.

Isopentenyl diphosphate (IPP) and its isoform dimethylallyl diphosphate (DMAPP), which are the common precursors of terpenoids, are produced *via* the mevalonate (MVA) and methylerythritol phosphate (MEP) pathways (Newman and Chappell, 1999; Querol et al., 2002). Although both pathways are thought to be compartmentally independent, there is some cross-talk between them (Laule et al., 2003; Dudareva et al., 2005). The MVA pathway occurs in the cytoplasm of most eukaryotes and is responsible for the synthesis of sesquiterpenes and triterpenes, whereas the MEP pathway occurs predominantly in eubacteria and the chloroplasts of photosynthetic eukaryotes to generate precursors for the synthesis of hormones, monoterpenes, and diterpenes (Rohdich et al., 2001; Dudareva et al., 2005). In the MEP pathway, 1-deoxy-D-xylulose 5-phosphate synthase (DXS) catalyzes the first reaction that converts pyruvate and glyceraldehyde-3-phosphate (GAP) to 1-deoxy-D-xylulose 5-phosphate (Lois et al., 1998; Querol et al., 2002). The MEP pathway subsequently produces IPP and DMAPP in a 5:1 ratio in six steps (Takahashi et al., 1998). There is experimental evidence that DXS is a rate-limiting enzyme and catalyzes an important step in the bottleneck of the pathway (Lange et al., 1998; Estevez et al., 2001). Unlike the other enzymes in the MEP pathway, which are mostly encoded by single-copy genes, DXS is usually encoded by multi-copy genes that are divided into three clades (Rodriguez-Concepcion and Boronat, 2002; Cordoba et al., 2009).

Previous studies on *DXS* focused on herbaceous plant species, including *Arabidopsis thaliana*, *Solanum lycopersicum*, *Zea mays*, and *Oryza sativa*. Of the three *DXS* genes identified in *A. thaliana*, only *AtDXS1* in clade 1 encodes a functional DXS protein (Carretero-Paulet et al., 2013). A mutation in *CLA1* (*DXS1*) disrupts normal chloroplast development and mesophyll tissue formation in *A. thaliana* (Estevez et al., 2000). Additionally, overexpression of *DXS1* in transgenic *A. thaliana* affects the accumulation of various isoprenoids such as chlorophylls, tocopherols, carotenoids, abscisic acid, and gibberellins (Estevez et al., 2001). The ectopic expression of *AtDXS1* in lavender increases the essential oil monoterpene content (Munoz-Bertomeu et al., 2006). Maize also carries three *DXS* genes, with each belonging to a different clade. Specifically, *DXS1* is highly expressed in photosynthetic tissues during early developmental stages, whereas *DXS2* is preferentially expressed in young seedling roots and may influence secondary metabolism (Cordoba et al., 2011). In contrast, the maize *DXS3* gene belongs to clade 3, but it has not been functionally characterized. Research regarding *S. lycopersicum* *DXS* genes revealed that *SlDXS1* is ubiquitously expressed, especially during the fruit-ripening stage, whereas *SlDXS2* is highly expressed in only a few tissues (Garcia-Alcazar et al., 2017). Furthermore, the non-redundant function of SlDXS2 involves modulating isoprenoid metabolism and the plasticity in the allocation of the isoprenoid precursor (Paetzold et al., 2010). A recent study confirms that OsDXS2 plays an important role as a rate-limiting enzyme supplying IPP/DMAPPs for the seed-carotenoid accumulation. The carotenoid metabolism in rice seed could be largely enhanced without any significant transcriptional alteration of carotenogenic genes (You et al., 2020). These findings imply that the *DXS* genes in clade 1 likely have housekeeping functions that are essential for plant growth and development, whereas those in clade 2 contribute to secondary metabolism (Saladie et al., 2014).

Substantial progress has been made in clarifying the roles of DXS in herbaceous plant species, but there has been relatively little research regarding DXS in woody plants. One exception is *Populus trichocarpa*. Specifically, earlier investigations proved that overexpression of *PtDXS* in transgenic lines increases abscisic acid and gibberellic acid (GA) contents, enhances resistance to *Septotis populiperda*, and decreases feeding by *Micromelalopha troglodyte* (Wei et al., 2019; Xu et al., 2019).

Mulberry is widely planted in the Eurasian continent and an important tree used for rearing the domesticated silkworm. The mulberry tree is also attractive to farmers with its tasty fruit and multiple uses in traditional Chinese medicine (He et al., 2013). There are multiple active compounds in mulberry, including ursolic acid, which is a terpenoid isolated from *Morus nigra* with antibacterial and anticancer properties (Chen et al., 2017). Terpenoids in mulberry are required for physiological activities and defense systems. Therefore, clarifying the mechanisms underlying terpenoid functions in mulberry is warranted. In this study, we conducted a series of experiments to analyze mulberry *DXS* genes. First, we identified three *MnDXS* genes and determined that *MnDXS1* belongs to clade 1, whereas *MnDXS2A* and *MnDXS2B* belong to clade 2. The encoded proteins were observed to localize in chloroplasts. Additionally, the expression patterns of the three *MnDXS* genes in various tissues and in response to silkworm feeding and methyl jasmonate (MeJA) treatment varied substantially. Transgenic lines overexpressing *MnDXS1* exhibited early flowering and increased GA accumulation, whereas overexpression of *MnDXS2A* positively influenced root growth and increased the chlorophyll and carotenoid content. These findings revealed the functional differences among the *MnDXS* genes.

## Materials and Methods

### Plant Materials and Treatments

Leaves, roots, stems, male flowers, female flowers, and seeds were collected from *Morus notabilis* trees growing in Ya’an city, Sichuan province, China. The collected samples were immediately frozen in liquid nitrogen and then stored at −80°C until used. The seeds were peeled and then germinated in agar-solidified half-strength Murashige and Skoog (MS) medium prepared with tap water until they formed roots. The resulting seedlings were transplanted into pots filled with peat moss and perlite (5:1). The plantlets were grown in a PQX plant incubator (Ningbo Southeast Instrument Corporation, Ningbo, China) at 25°C with a 12-h light/12-h dark photoperiod until the aerial parts grew to approximately 25 cm.

Seedling leaves were treated with 2- or 3-d-old fifth instar silkworm larvae or were sprayed with 0.1 mM MeJA or with water. The seedling leaves without treatment were used as control check (CK). The seedlings were incubated for 2 h, after which the leaves were collected, immediately frozen in liquid nitrogen, and then stored at −80°C for a total RNA extraction. Untreated leaves were used as controls. All experiments were carried out in three independent biological replicates.

### RNA Extraction and Quantitative Real-Time (qRT)-PCR Analysis

Total RNA was extracted from the collected mulberry roots, stems, male flowers, female flowers, and leaves as well as the stress-treated leaves with the RNAiso Plus Kit (Takara Bio., Shiga, Japan). Total RNA (1 μg) was used as the template to synthesize cDNA with the PrimeScript^™^ RT Reagent Kit (Perfect Real Time) (Takara Bio., Shiga, Japan). A qRT-PCR assay was completed with SYBR^®^ Premix Ex Taq^™^ II (Takara Bio., Shiga, Japan). The *CTR9* gene served as the reference control for normalizing CT values. Gene-specific qRT-PCR primers were designed with Primer Premier 5 (**Table S1**). The 2^−ΔΔCT^ method was used to calculate the relative fold-changes in gene expression (Livak and Schmittgen, 2001). The qRT-PCR was carried out in three independent technical replicates.

### Cloning of DXS Genes and Phylogenetic Analysis

The *AtDXS1*, *AtDXS2*, and *AtDXS3* sequences were used as queries to screen the *M. notabilis* genome database with the BLAST algorithm to identify *MnDXS* genes (http://morus.swu.edu.cn/morusdb/). The cDNA prepared from *M. notabilis* leaves was used as the templates to clone genes with Ex Taq DNA Polymerase (Takara Bio., Shiga, Japan). The PCR primer pairs for amplifying specific coding sequences were designed based on the available *MnDXS1, MnDXS2A*, and *MnDXS2B* sequences (**Table S1**). The PCR conditions were as follows: 94°C for 4 min; 32 cycles of 94°C for 30 s, 58°C for 30 s, and 72°C for 2.5 min; 72°C for 7 min. The amplified *DXS* genes were cloned into the pMD19-T vector (TaKaRa, Dalian, China) and sequenced to verify accuracy. A phylogenetic tree based on the DXS protein sequences from plants, algae, and bacteria was constructed according to the neighbor-joining method of MEGA 7, with 1,000 bootstrap replicates. The *Deinococcus radiodurans*, *Escherichia coli*, and *Aegilops variabilis* sequences were used as references. The phylogenetic tree was drawn with iTOL (https://itol.embl.kde).

### Subcellular Localization

The putative plastid transit peptides of DXS isoforms were predicted with TargetP 1.1. The fragments of three *DXS* genes amplified with specific primers were inserted into the *Bam*HI site of the pTF486 expression vector, which includes an enhanced green fluorescent protein (EGFP) coding sequence (**Table S1**). For each recombinant plasmid, approximately 2 μg was introduced into *A. thaliana* mesophyll protoplasts *via* polyethylene glycol-mediated transformation (Yoo et al., 2007). The EGFP fluorescence and chlorophyll autofluorescence were observed with the FV1200 confocal laser scanning microscope (Olympus, Tokyo, Japan) at excitation wavelengths of 488 and 561 nm, respectively.

### Construction of the *MnDXS1*, *MnDXS2A* and *MnDXS2B* Overexpression Plasmids

The full-length *MnDXS1, MnDXS2A*, and *MnDXS2B* coding sequences were amplified by PCR. They were then inserted between the *Bam*HI and *Eco*RI sites of the pLGNL expression vector under the control of the cauliflower mosaic virus 35S promoter and followed at the 3′ end by the nopaline synthase gene terminator. The primers used for constructing the recombinant plasmids were listed in **Table S1**.

### Transformation and Analysis of Transgenic *Arabidopsis* *thaliana*

The *MnDXS1*, *MnDXS2A*, and *MnDXS2B* overexpression plasmids were inserted in wild-type (WT) *A. thaliana* protoplasts (ecotype Columbia) *via A. tumefaciens*-mediated plant transformation involving the floral dipping method (Clough and Bent, 1998). Transformed seedlings identified on agar-solidified selective medium containing 50 mg/L kanamycin were transferred to soil. Plants were incubated in a growth chamber at 24°C under a 16-h light/8-h dark photoperiod. Finally, the homozygous transgenic *A. thaliana* lines from the T_3_ generation were analyzed further.

The WT and homozygous transgenic *A. thaliana* seedlings were grown on agar-solidified half-strength MS medium. The seedlings were incubated for 8 d in a growth chamber at 23°C, with a 16-h light/8-h dark photoperiod. The total root length of the seedlings was measured with the WinRHIZO Arabidopsis system. The root length measurement was performed on three independent biological replicates. After transferring the seedlings to soil, the time to flowering for six well-growing plants from each transgenic line was determined and calculated as the time from when the seedlings were transferred to soil until bolting to 1 cm. A qRT-PCR assay was carried out to assess the influence of *MnDXS* overexpression on the flowering time gene *SOC1* (suppressor of overexpression of CO1). The *AtActin2* gene was used as the internal control to normalize the expression levels. The leaves from the well-growing plants were used for RNA extraction and the qRT-PCR was carried out in three independent technical replicates. Total RNA isolation, reverse transcription, and qRT-PCR methods were completed as described previously.

The fresh leaves of WT plants and *MnDXS1*, *MnDXS2A*-overexpressing transgenic lines were collected for the measurement of chlorophyll and carotenoid using the Plant Chlorophyll and Carotenoid Content Kit (Cao Benyuan, Nangjing, China) by microplate method and the measurements were carried out in three independent biological replicates. The extracted solution of leaves was measured of absorbance at 470, 645, and 663 nm. The absorbance value of the extracting solution (acetone: ethyl alcohol = 2: 1) was used for CK. The corresponding pigment content was calculated according to the following formula: chlorophyll A (mg/g) = 0.02 × (12.7 × A663 − 2.69 × A645) × D ÷ M; chlorophyll B (mg/g) = 0.02 × (22.9 × A645 − 4.68 × A663) ×  D ÷ M; total chlorophyll (mg/g) = 0.02 × (20.21 × A645 + 8.02 × A663) × D ÷ M; carotenoid (mg/g) = 0.02 × ((1000 × A470 − 3.27Ca − 104Cb) ÷ 229) × D ÷ M. V stands for the volume of extracting solution, D stands for the dilution factor, and m stands for the weight of collected sample.

The stems and leaves of WT plants and *MnDXS1* and *MnDXS2A*-overexpressing transgenic lines were collected at same position to measure the GA content using the Plant Gibberellic Acid ELISA Kit (Cao Benyuan, Nangjing, China). In addition, the whole plants were used for measuring abscisic acid (ABA) content with the Plant Hormone ABA ELISA Kit (Cao Benyuan, Nangjing, China). The measurements were carried out in three independent biological replicates. Double antibody sandwich method was used to determine the GA and ABA content in plants. Blank wells, standard wells, and sample wells were set for the determination of content. The blank wells were used for CK. In the experiment for determining the GA content, the concentration of standard solution was 0, 3, 6, 12, 24, and 48 nmol/L. According to the absorbance value at 450 nm of standard solution and its corresponding concentration, a standard curve (y = 0.0278x + 0.032, R^2^ = 0.9978) was produced. The GA content in tested samples was then calculated according to this formula. While in determining the ABA content, the standard solution in 0, 5, 10, 20, 40, and 80 ng/ml was used for producing the standard curve (y = 0.0142x − 0.0266, R^2^ = 0.9879). According to the formula, the ABA content was calculated.

### qRT-PCR Analysis of Biosynthesis-Related Genes

The relative expression levels in A. *thaliana* of *geranyl (geranyl) diphosphate synthase 11 (GGPPS11)* for chlorophyll biosynthesis, *phytoene synthase (PSY)* for carotenoid biosynthesis, *ent-copalyl diphosphate synthase (CPS)*, and *entkaurene synthase (KS)* for GA biosynthesis and *nine-cis-epoxycarotenoid dioxygenase 2 (NCED2)* for ABA biosynthesis of WT plants and MnDXS1, *MnDXS2A*-overexpressing transgenic lines were determined using qRT-PCR. Total RNA was extracted from the collected transgenic *A. thaliana* leaves, stems, and roots. The expression patterns of chlorophyll, carotenoid, GA, and ABA metabolism-related genes were analyzed using the RNAiso Plus Kit (Takara Bio., Shiga, Japan). All experiments were carried out in three independent biological replicates. Total RNA (1 μg) was used as the template to synthesize cDNA with the PrimeScript™ RT Reagent Kit (Perfect Real Time) (Takara Bio., Shiga, Japan). A qRT-PCR assay was completed with SYBR^®^ Premix Ex Taq^™^ II (Takara Bio., Shiga, Japan). The *AtActin2* gene was used as the internal control to normalize the expression levels. Gene-specific qRT-PCR primers were designed with Primer Premier 5 (**Table S1**). The 2^−ΔΔCT^ method was used to calculate the relative fold-changes in gene expression (Livak and Schmittgen, 2001). The qRT-PCR was carried out in three independent technical replicates.

## Results

### Three Mulberry DXS Genes

**Figure 1:**
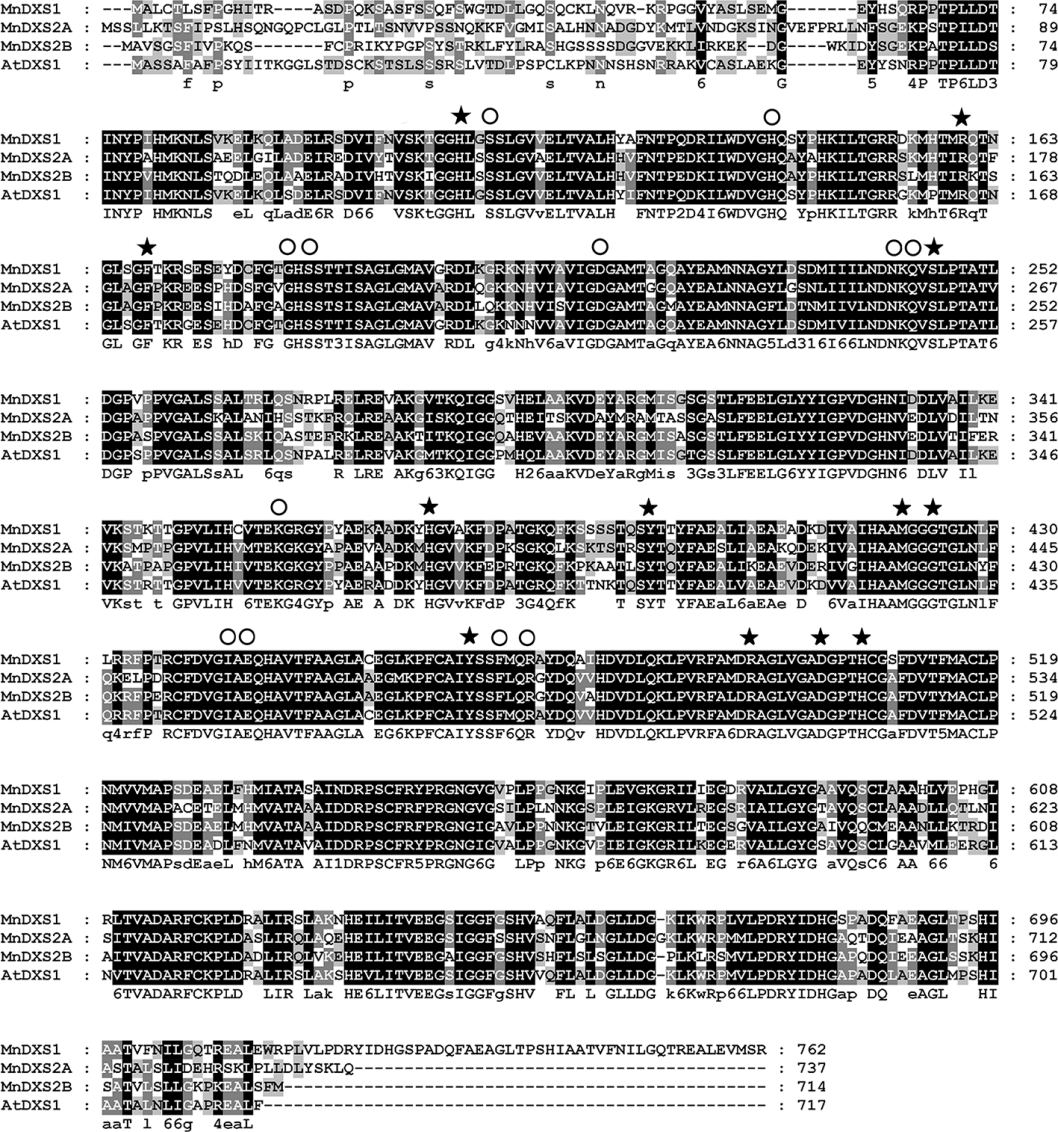
Alignment of the MnDXS1, MnDXS2A, and MnDXS2B amino acid sequences with the *Arabidopsis thaliana* DXS1 protein. Residues in the (GAP-binding and TPP-binding domains are marked with solid stars and circles, respectively.

The three *MnDXS* genes identified in the *M. notabilis* genome database, Morus008477, Morus022865, and Morus026786 were named as *MnDXS1*, *MnDXS2A*, and *MnDXS2B*, respectively. The *MnDXS* cDNA sequences were 2,289 (*MnDXS1*), 2,214 (*MnDXS2A*), and 2,145 bp (*MnDXS2B*) long. Additionally, the encoded amino acid sequences were more than 65% identical, with conserved GAP-binding and TPP-binding domains (**Figure 1**).

**Figure 2:**
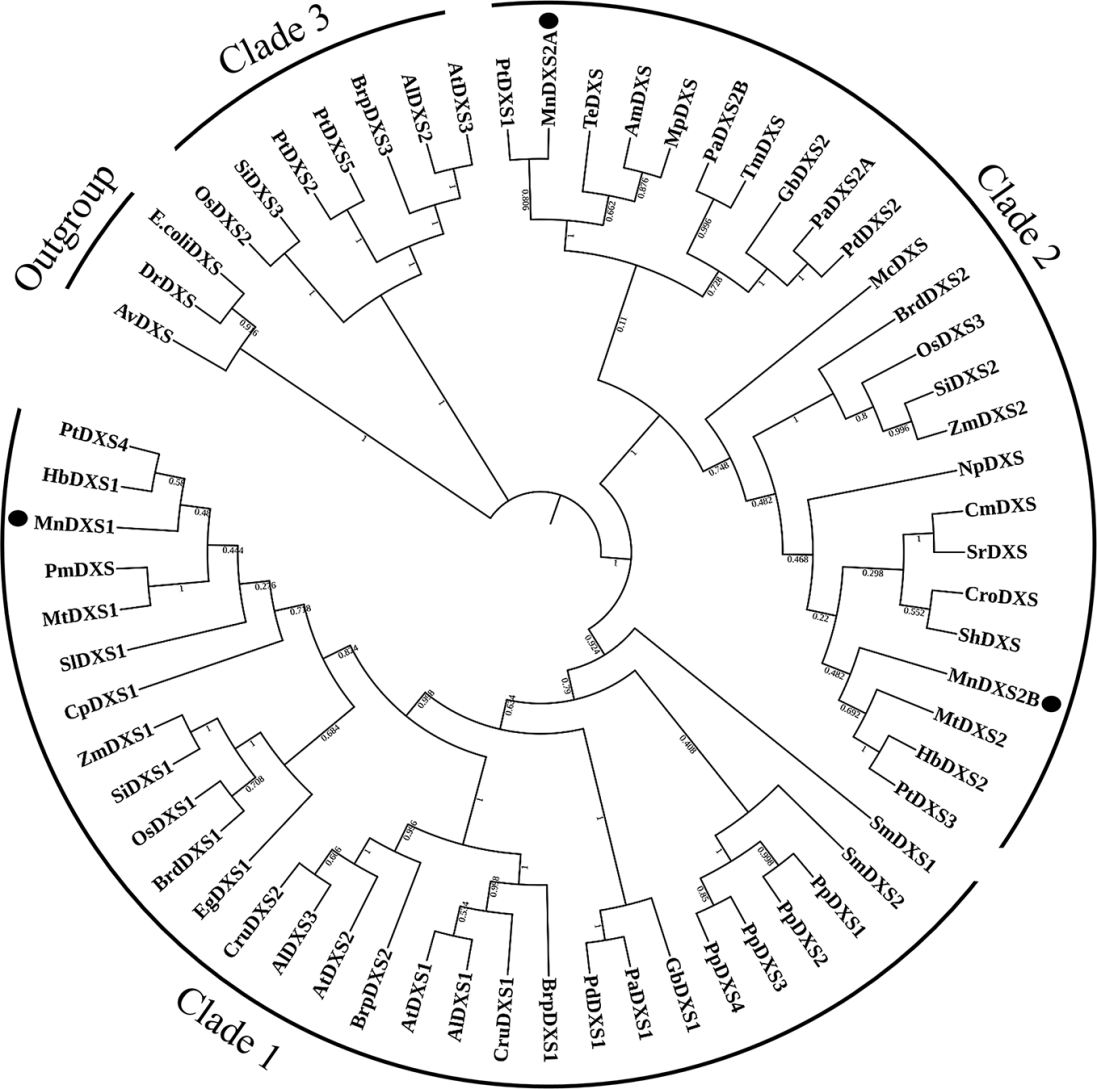
Phylogenetic relationships among DXS proteins. The National Center for Biotechnology Information (NCBI) accession numbers of the amino acid sequences included in the phylogenetic tree are listed in **Table S2**.

The constructed phylogenetic tree revealed that MnDXS1 belongs to clade 1, which includes the well-characterized DXS1 proteins from *A. thaliana*, *Z. mays*, and other plant species (**Figure 2**). Clade 2 comprised MnDXS2A, MnDXS2B, and representatives from *Medicago truncatula*, *Catharanthus roseus*, and other species.

### Subcellular Localization of MnDXS Proteins in Protoplasts

**Figure 3:**
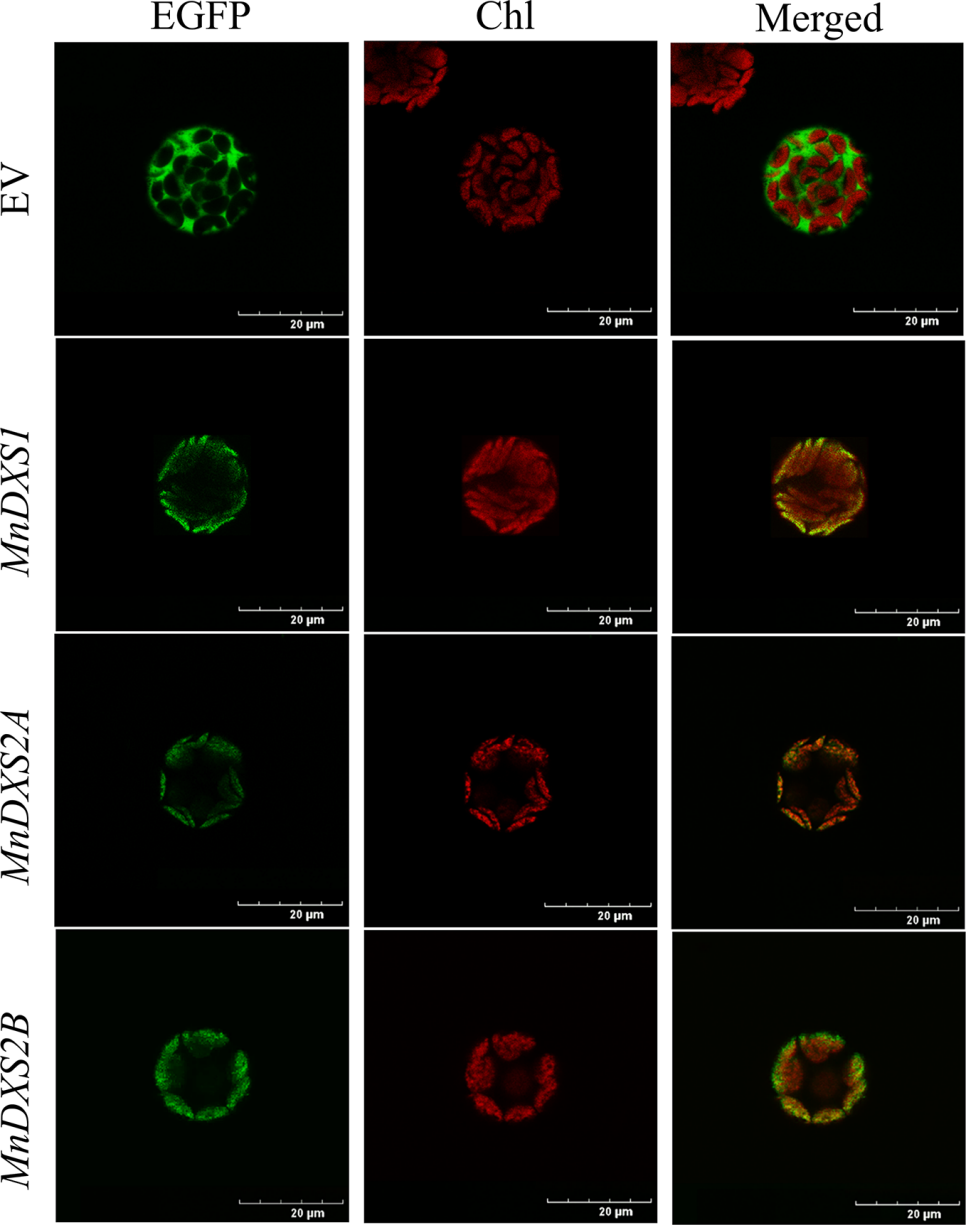
Images of *Arabidopsis thaliana* mesophyll protoplasts producing MnDXS-EGFP fusion proteins. The EGFP fluorescence and chlorophyll autofluorescence were detected by confocal laser scanning microscope.

An analysis with TargetP 1.1 predicted the three MnDXS proteins are most likely localized in chloroplasts. The subcellular localization based on an examination of fluorescence by confocal laser scanning microscopy produced consistent results (**Figure 3**). Specifically, the EGFP signal overlapped with the chlorophyll autofluorescence signal. Because the MEP pathway is a plastid pathway, it is reasonable that these rate-limiting enzymes are localized in chloroplasts.

### Expression Analysis of the *MnDXS* Genes in Mulberry Tissues and in Response to Stress

**Figure 4:**
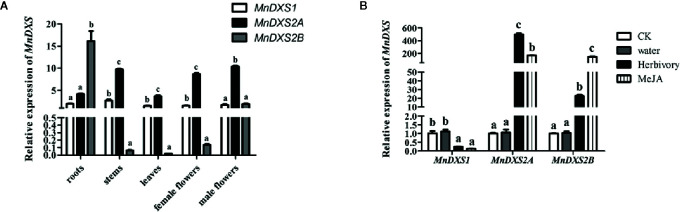
Relative *MnDXS* expression levels in various tissues and in response to stress treatments based on a qRT-PCR assay. The *Morus notabilis CTR9* gene was used as the internal control to normalize the expression levels. **(A)** Relative *MnDXS1*, *MnDXS2A*, and *MnDXS2B* expression levels in the roots, stems, leaves, female flowers, and male flowers. **(B)** Relative *MnDXS* expression levels in leaves in response to silkworm feeding, MeJA and water. Data are presented as the mean ± standard error the mean of three replicates. The analysis of significant differences was conducted for each gene. CK stands for control without any treatment. Different letters above bars represent significant differences (p < 0.05) as determined with the one-way Duncan’s test.

The qRT-PCR assay revealed diverse *MnDXS* expression profiles among the examined tissues (**Figure 4A**). The *MnDXS1* and *MnDXS2A* genes were similarly expressed at relatively high levels in all tissues. In contrast, although *MnDXS2B* was the most highly expressed gene in the roots, it was expressed at very low levels in the stems, leaves, and female flowers. The stress treatments affected the *MnDXS1* expression level differently than the *MnDXS2A* and *MnDXS2B* expression levels (**Figure 4B**). The 2-h feeding by silkworm larvae and MeJA treatment tended to downregulate *MnDXS1* expression, whereas these stresses had the opposite effect on *MnDXS2A* and *MnDXS2B*.

### Overexpression of *MnDXS* Genes Altered *Arabidopsis thaliana* Phenotypes

A comparison of the root lengths of WT and *MnDXS*-overexpressing transgenic lines revealed the genes differentially affected root growth (**Figure S2**). In agar-solidified half-strength MS medium, the roots of WT and *MnDXS1*-overexpressing lines were 32–35 mm long, whereas the roots of *MnDXS2A*-overexpressing plants were 40–56 mm long.

**Figure 5:**
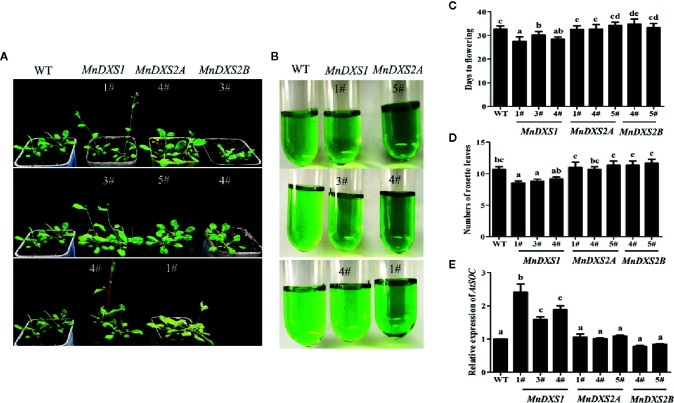
Days to flowering, the number of rosette leaves, and the pigment content for the WT and transgenic *A. thaliana* plants. **(A)** Phenotypes of 30-d-old wild-type and *MnDXS1*-, *MnDXS2A*-, and *MnDXS2B*-overexpressing transgenic *A. thaliana* plants. **(B)** The pigment extracted solution of WT and transgenic *A. thaliana* plants. **(C, D)** Days to flowering, the number of rosette leaves for the WT and transgenic *A. thaliana* plants. **(E)** qRT-PCR analyses of relative *AtSOC1* expression levels in WT and transgenic *A. thaliana* plants based on a qRT-PCR assay, with *Actin2* used as the internal control. Data are presented as the mean ± standard error of the mean of three replicates. Different letters above bars represent significant differences (p < 0.05) as determined with the one-way Duncan’s test.

The *MnDXS1*-overexpressing transgenic lines flowered earlier and produced fewer rosette leaves than the WT plants (**Figures 5A–D**). Moreover, the *SOC1* expression level was higher in the *MnDXS1*-overexpressing transgenic lines than those in the WT plants and other transgenic lines (**Figure 5E**). In *MnDXS2A*-overexpressing lines, greener leaves and better growth were observed (**Figures 5A, B**).

**Figure 6:**
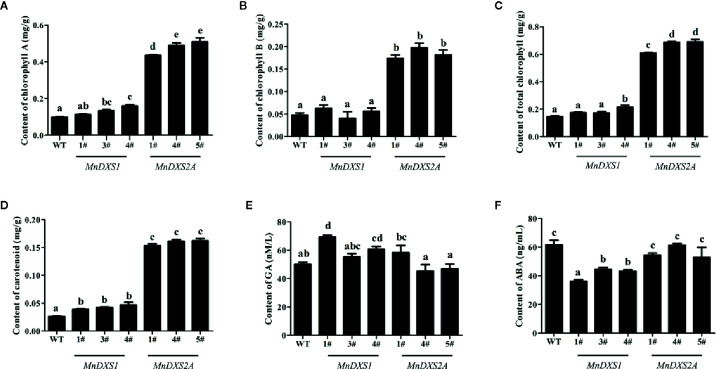
Chlorophyll **(A–C)**, carotenoid **(D)**, GA **(E)**, and ABA **(F)** content in WT plants and *MnDXS1*- *MnDXS2A*-overexpressing transgenic lines. Data are presented as the mean ± standard error of the mean of three replicates. Different letters above bars represent significant differences (p < 0.05) as determined with the one-way Duncan’s test.

As shown in **Figures 6A–D**, the total chlorophyll (including chlorophyll A and B) and carotenoid content were higher in *MnDXS2A* transgenic lines than those of WT plants and *MnDXS1*-overexpressing lines. The GA content of *MnDXS1*-overexpressing transgenic lines was significantly higher than that of WT plants, while the ABA content was decreased (**Figures 6E, F**). However, the GA content in different *MnDXS2A* transgenic lines displayed divergent trends compared with WT plants and the ABA content was slightly decreased (**Figures 6E, F**).

### Overexpression of *MnDXS1* and *MnDXS2A* Changed Relative Expression Levels of Biosynthesis-Related Genes

**Figure 7:**
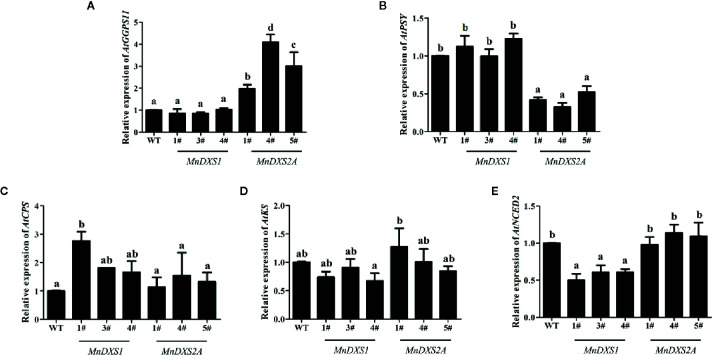
Relative expression levels of biosynthesis-related genes- *geranyl (geranyl) diphosphate synthase 11 (GGPPS11)* for chlorophyll synthesis **(A)**, *phytoene synthase (PSY)* for carotenoid synthesis **(B)**, *ent-copalyl diphosphate synthase (CPS) and entkaurene synthase (KS)* for GA synthesis **(C, D)**, and *nine-cis-epoxycarotenoid dioxygenase 2 (NCED2)* for ABA synthesis **(E)** in WT plants and *MnDXS1*-, *MnDXS2A*-overexpressing transgenic lines. The *Actin2* gene was used as the internal control to normalize the expression levels. Data are presented as the mean ± standard error of the mean of three replicates. Different letters above bars represent significant differences (p < 0.05) as determined with the one-way Duncan’s test.

As shown in **Figure 7**, in the *MnDXS1*-overexpressing lines, the expression levels of *AtKS* and *AtNCED2* were downregulated to 76% and 57% compared with the WT plants. The relative expression levels of *AtGGPPS11* and *AtPSY* were kept at the same levels as the WT plants, while those of *AtCPS* was upregulated to 220%. In the *MnDXS2A*-overexpressing lines, the expression levels of *AtGGPPS11* were upregulated to 300%, while those of *AtPSY* were downregulated to 42%. The relative expression levels of *AtCPS*, *AtKS*, and *AtNCED2* were maintained at the same levels as the WT plants.

## Discussion

The MEP pathway generates IPP and DMAPP, which are precursors for the biosynthesis of hormones, monoterpenes, and diterpenes (Chappell, 2002). Terpenoids are necessary for plant growth and development and responses to environmental factors. They are also useful for humans. Therefore, the mechanisms underlying plant terpenoid metabolism and regulation will need to be more thoroughly characterized. In the MEP pathway, DXS catalyzes the first and the rate-limiting reaction, and mutations to the DXS-encoding genes in *A.* *thaliana* reportedly result in an albino phenotype (Estevez et al., 2000). A recent report suggested DXS may be an important target in *Nicotiana benthamiana* for manipulating the biosynthesis of taxanes, which are diterpenoids that have anticancer properties (Li et al., 2019). Previous studies on DXS confirmed that it is an essential enzyme in plants and a key target for manipulating certain tepenoids. Mulberry is a woody plant species abundant in metabolites that may be useful for treating diseases. In this study, we demonstrated that the *MnDXS* genes from different clades have non-redundant functions. Additionally, overexpression of *MnDXS* influences plant development and metabolism.

An analysis of three *M. notabilis DXS* genes uncovered sequences encoding conserved TPP-binding and GAP-binding domains, which are necessary for the biosynthesis of thiamine (vitamin B1) and pyridoxol (vitamin B6) (Xiang et al., 2007). Although the three MnDXS proteins are more than 65% similar regarding their amino acid sequences and are localized in chloroplasts, the corresponding genes belong to different clades. Specifically, *MnDXS1* is in clade 1, whereas *MnDXS2A *and *MnDXS2B* belong to clade 2. These genes were differentially expressed in various mulberry tissues. More importantly, stresses imposed by silkworm feeding and MeJA treatments decreased *MnDXS1* expression levels, but considerably increased *MnDXS2A* and *MnDXS2B* expression levels. We hypothesized that the upregulated expression of *MnDXS2A* and *MnDXS2B* might be related to certain metabolites involved in the plant defense system against herbivores. Additionally, MnDXS1 likely has a housekeeping function. Analyses of *MnDXS*-overexpressing lines supported our hypothesis that different *MnDXS* genes from different clades had distinct function. Surprisingly, the *MnDXS1*-overexpressing transgenic lines unexpectedly flowered earlier than the WT plants. Moreover, overexpression of *MnDXS2A* induced root growth. We speculated that overexpression of *MnDXS2A* may increase the abundance of specific terpenoids secreted from the roots to soil and further promote the absorption of nutrients by the roots.

It has been reported that the carotenoid, chlorophyll, ABA, and GA are derived from the common precursor geranylgeranyl diphosphate (GGPP) (Moreno et al., 2016). Overexpression of *MnDXS* genes belonging to different clades contributed to distinct content of these substances. The total chlorophyll content (including chlorophyll A and B) and carotenoid content were both significantly higher in *MnDXS2A*-overexpressing transgenic lines than those in WT and *MnDXS1*-overexpressing plants. The highest levels of GA content were observed in *MnDXS1*-overexpressing lines while the ABA content was obviously decreased. Although GGPP is the common precursor, it seemed that *MnDXS2A* was involved in the synthesis of chlorophyll and carotenoid, and overexpression of *MnDXS1* contributed to the GA accumulation. The functions of *MnDXS* genes were differentiated.

The high levels of GA content were observed in the *MnDXS1*-overexpressing lines. The increased GA content may result in early flowering. A previous study on *A. thaliana* identified the following five ﬂowering-time pathways: age, autonomous, gibberellin, photoperiod, and vernalization (Amasino and Michaels, 2010). GA is a tetracyclic diterpene growth factor with an essential role in many aspects of plants development, including seed germination, stem and petiole elongation, floral induction, and floral organ development. Experimental evidence also suggests that the GA biosynthesis is correlated with the MEP pathway (Yamaguchi and Kamiya, 2000). The fact that the GA content was highest in the *MnDXS1*-overexpressing transgenic lines is consistent with our hypothesis that higher GA content could lead to earlier flowering. A flowering-related function for DXS was detected in an earlier investigation involving rose petals, in which *DXS* gene expression levels increased from the bud to blooming stages (Feng et al., 2014). Additionally, in the gibberellin synthesis pathway, the CPS and KS catalyze GGDP to ent-kaurene, and CPS is a rate-limiting enzyme that determines the level of GGDP converted into gibberellins (Huang et al., 2012). It was reported that overexpression of *AtCPS* in *A. thaliana* results in high levels of ent-kaurene, but overexpression of *AtKS* does not increase the ent-kaurene content, indicating that they have different regulation mechanisms (Fleet et al., 2003). In the present study, *AtCPS* was higher expressed in *MnDXS1* transgenic lines with higher GA content. The relative expression levels of *GGPPS11* involved in chlorophyll synthesis were upregulated in *MnDXS2A* transgenic lines and those of *AtNCED2* involved in ABA synthesis were downregulated in *MnDXS1* lines. However, the expression profile of the gene involved in carotenoid synthesis was not consistent to the content variation. It is reasonable that the transcriptional expression of the intrinsic metabolism gene (*AtPSY*) might not be proportional to the expression levels of their encoded-proteins. And the protein stability of the intermediate biosynthetic enzymes might be equally as important as the enhanced activity of rate-limiting enzymes for the content enhancement (You et al., 2020).

The data presented herein prove that the *MnDXS* genes in different clades are functionally diverse and have non-redundant roles. The clade 1 gene *MnDXS1* influences the GA content and the flowering process. Moreover, to the best of our knowledge, this is the first study to observe an early-flowering phenotype during a functional analysis of DXS. The clade 2 *MnDXS* genes participate in defense mechanisms and are related to root development, which are associated with secondary metabolism (Floss et al., 2008). And the chlorophyll and carotenoid content significantly increase in *MnDXS2A*-overexpressing lines. Although our findings are important for the molecular characterization of *MnDXS* genes, there remain unresolved issues. For example, we determined that *MnDXS* genes encoding similar amino acid sequences have distinct functions, but the underlying mechanisms remain unclear. Also the heterologous expression of *MnDXS* genes in *A. thaliana* was not sufficient enough to elucidate the function of *MnDXS* genes. Nevertheless, our results may be useful for future research regarding *DXS* genes in mulberry and related species.

## Conclusion

We cloned three *MnDXS* genes and revealed the functional diversity among these genes. Overexpression of *MnDXS1* in *A. thaliana* increased the GA content and resulted in early flowering. The *MnDXS* genes from clade 2 might mediate the synthesis of terpenoids related to plant defenses against herbivores. Additionally, overexpression of *MnDXS2A* resulted in higher chlorophyll and carotenoid content and promoted root growth. In conclusion, diverse DXS types influence various physiological activities in plants. These results may provide new perspectives for further research on DXS in *M. notabilis* and related plant species.

## Data Availability Statement

The NCBI accession numbers for the three MnDXS genes are XM_024171193.1, XM_010104684.2, and XM_010115150.2.

## Author Contributions

SZ, GD, and NH conceived and designed the study. GD performed the bioinformatics analysis. KL and JT did the RNA extraction and qRT-PCR analysis. SZ and WH performed the plant transformation and subcellular localization. SZ and WH managed *Arabidopsis*. SZ and GD analyzed the data. YL provided the technical assistance. SZ wrote the manuscript and NH revised the manuscript. All authors contributed to the article and approved the submitted version.

## Funding

This project was funded by the National Key R&D Program of China (No. 2019YFD1001200), Natural Science Foundation of China (No. 31572323), and Fundamental Research Funds for the Central Universities (XDJK2019C012 and XDJK2019C088).

## Conflict of Interest

The authors declare that the research was conducted in the absence of any commercial or financial relationships that could be construed as a potential conflict of interest.
