# Supplementary figures and images for: Functional Characterization of the 1-Deoxy-D-Xylulose 5-Phosphate Synthase Genes in Morus notabilis
Source: Front Plant Sci. 2020 Jul 24;11:1142. doi: 10.3389/fpls.2020.01142 (PMC7396507; doi:10.3389/fpls.2020.01142)

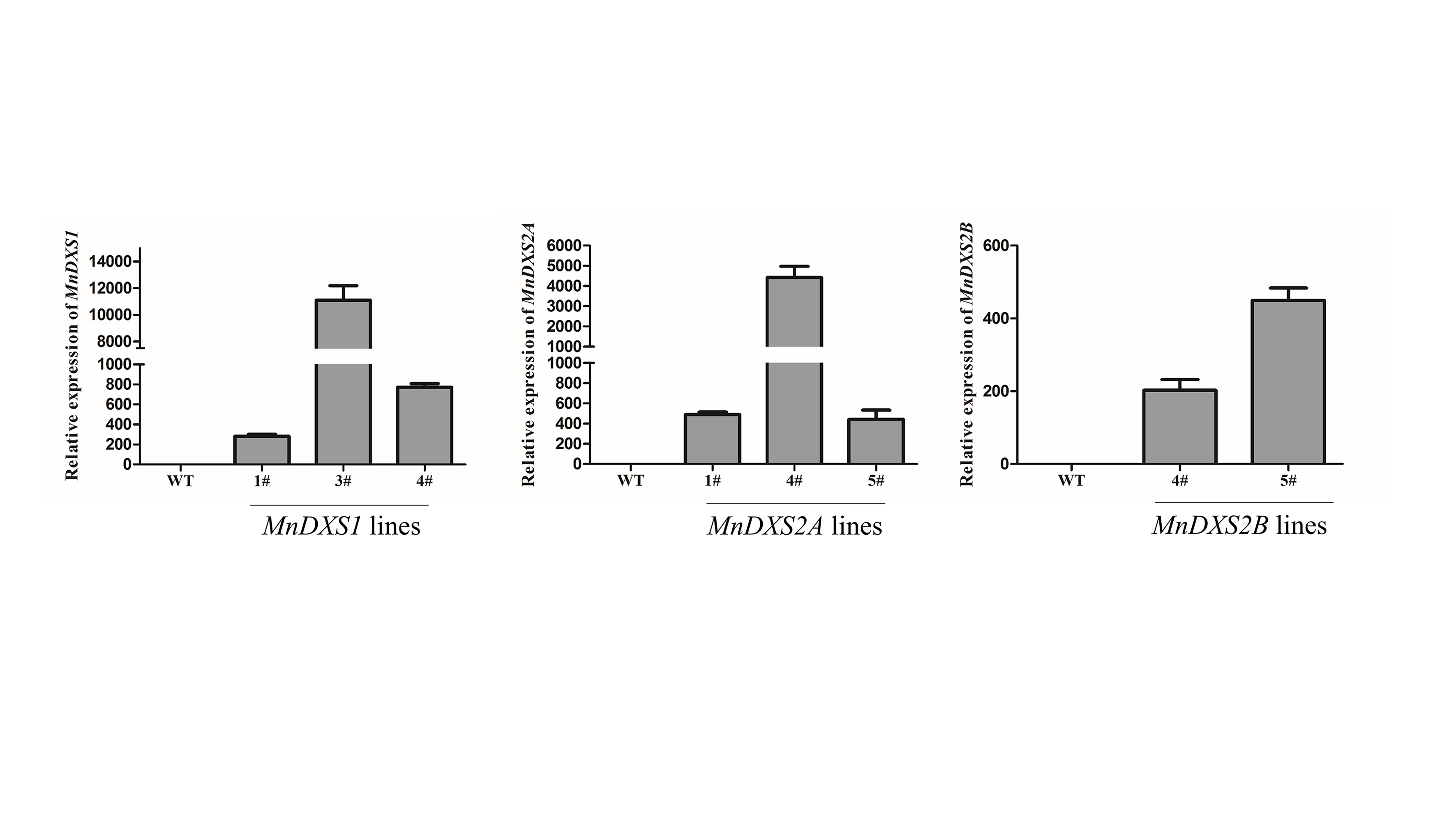

Supplement: Supplementary file 1 [file Image_1.tif]

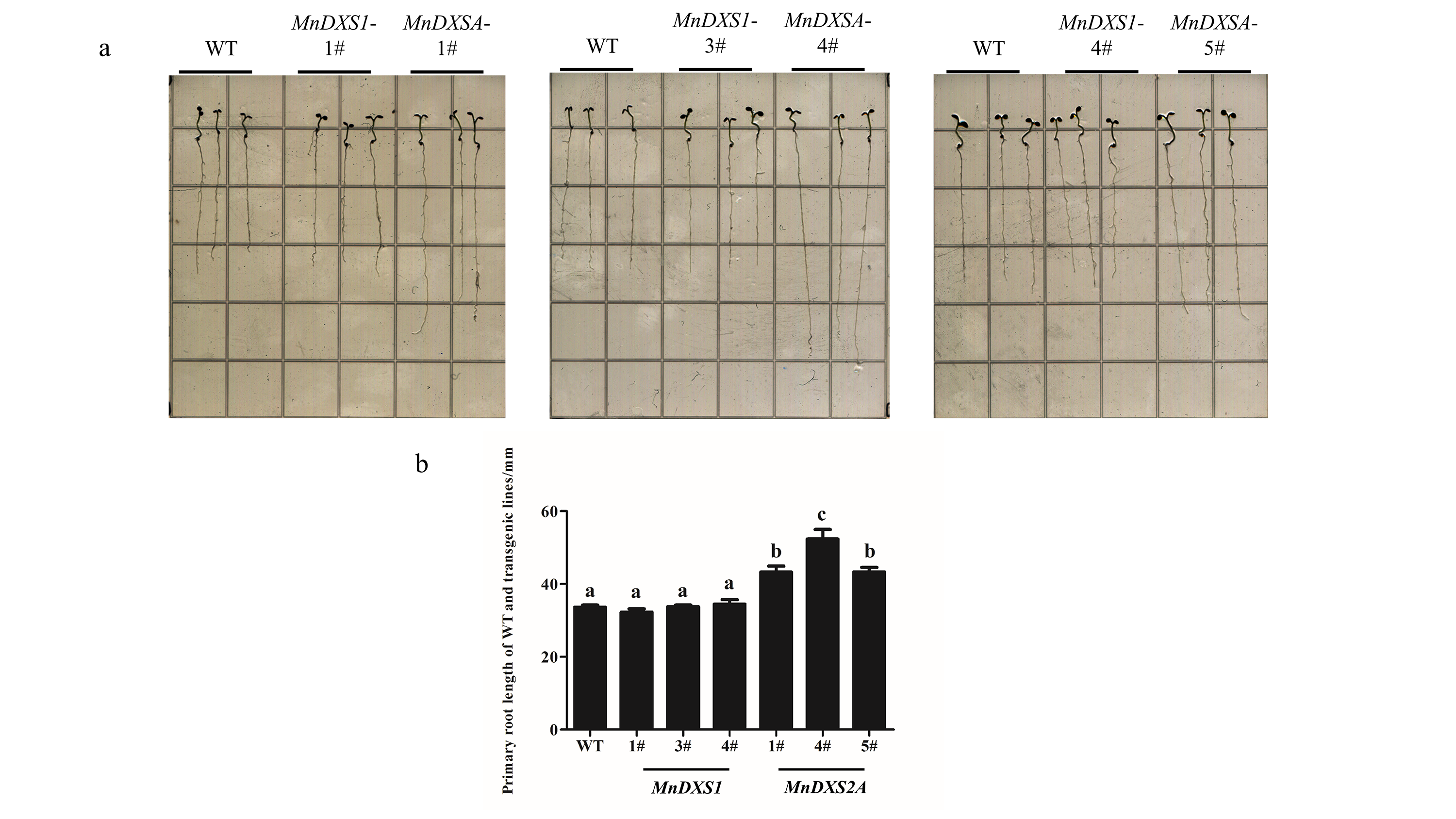

Supplement: Supplementary file 2 [file Image_2.tif]
