# Supplementary material for: Functional Characterization of the 1-Deoxy-D-Xylulose 5-Phosphate Synthase Genes in Morus notabilis
Source: Front Plant Sci. 2020 Jul 24;11:1142. doi: 10.3389/fpls.2020.01142 (PMC7396507; doi:10.3389/fpls.2020.01142)
Supplement: Supplementary file 3 [file Table_1.docx]

Table S1. Sequences of the primers used in this study

| Primer name | Primer sequence |
| --- | --- |
| MnDXS1-F | 5'-ATGGCGCTTTGTACATTATCGT-3' |
| MnDXS1-R | 5'-TCATCGTGACATGACTTCAAGA-3' |
| MnDXS2A-F | 5'-ATGTCTTCTCTTCTCAAAACTAGTT-3' |
| MnDXS2A-R | 5'-CTACTGTAATTTTGAATACAGGTCG-3' |
| MnDXS2B-F | 5'-ATGGCGGTTTCTGGGTCTTTC-3' |
| MnDXS2B-R | 5'-TCACATGAAAGAAAGTGCTTCCTTG-3’ |
| MnDXS1-qF | 5'-ATGGCGCTTTGTACATTATCGT-3' |
| MnDXS1-qR | 5'-TCATCGTGACATGACTTCAAGA-3’ |
| MnDXS2A-qF | 5’-CCCTCGCCAATATTCACTCT-3’ |
| MnDXS2A-qR | 5’-CTGAACTGGCTGTCATTGCT-3’ |
| MnDXS2B-qF | 5’-CATCATTCCTGCAAAGAGGA-3’ |
| MnDXS2B-qR | 5’-CAGGCCATGTAGGTGACATC-3’ |
| Ctr9-qF | 5’-GGCTATGTGATTTACCGTGTT-3’ |
| Ctr9-qR | 5’-TTGGTCCAGTATGAGTTGAGA-3’ |
| AtGGPPS11-qF | 5’- TCGCCGGTGGCAAAAGAGTT -3’ |
| AtGGPPS11-qR | 5’- TCAGAACTCGTCGCCGAAGC -3’ |
| AtPSY-qF | 5’- TCTATTGTGGCTCTTGTTTGGTG -3’ |
| AtPSY-qR | 5’- CGAAAGGACGACCACGGAAA -3’ |
| AtNCED2-qF | 5’- GCACGGTGCAGATCGACGTA -3’ |
| AtNCED2-qR | 5’- GGTTTCGCCGGACGGAAGAT -3’ |
| AtCPS-qF | 5’-GGGGCAATCAAACCAAGCAG-3’ |
| AtCPS-qR | 5’- CGTCTCTACTCGAGGCAAGC-3’ |
| AtKS-qF | 5’- AGCCGAGTGGTCCAGTGACA-3’ |
| AtKS-qR | 5’- GCTTCCCTTCCGCGCTTTCT- 3’ |
| MnDXS1-  pLGNL-F | 5’-CAGGGTACCCGGGGATCCATGGCGCTTTGTACATTATCG-3’ |
| MnDXS1-  pLGNL-R | 5’-CTCATTAAAGCAGGGAATTCTCATCGTGACATGACTTCAAG-3’ |
| MnDXS2A-  pLGNL-F | 5’-CAGGGTACCCGGGGATCCATGTCTTCTCTTCTCAAAACTAG-3’ |
| MnDXS2A-pLGNL-R | 5’-CTCATTAAAGCAGGGAATTCCTACTGTAATTTTGAATACAGGTC-3’ |
| MnDXS2B-pLGNL-F | 5’-CAGGGTACCCGGGGATCCATGGCGGTTTCTGGGT-3’ |
| MnDXS2B-pLGNL-R | 5’-CTCATTAAAGCAGGGAATTCTCACATGAAAGAAAGTGCTTCC-3’ |
| MnDXS1-  pTF486-F | 5’-TTACTATTTACAATTACAGTCGACATGGCGCTTTGTACATTATC-3’ |
| MnDXS1- pTF486-R | 5’-ACGATCTGCAGCCGGGCGGCCGCTCGTGACATGACTTCAAGA-3’ |
| MnDXS2A-pTF486-F | 5’-TTACTATTTACAATTACAGTCGACATGTCTTCTCTTCTCAAAAC-3’ |
| MnDXS2A-pTF486-F | 5’-ACGATCTGCAGCCGGGCGGCCGCCTGTAATTTTGAATACAGG-3’ |
| MnDXS2B-pTF486-F | 5’-TTACTATTTACAATTACAGTCGACATGGCGGTTTCTGGGTCTT-3’ |
| MnDXS2B-pTF486-R | 5’-ACGATCTGCAGCCGGGCGGCCGCCATGAAAGAAAGTGCTTCC-3’ |
