# Supplementary material for: Functional Characterization of the 1-Deoxy-D-Xylulose 5-Phosphate Synthase Genes in Morus notabilis
Source: Front Plant Sci. 2020 Jul 24;11:1142. doi: 10.3389/fpls.2020.01142 (PMC7396507; doi:10.3389/fpls.2020.01142)
Supplement: Supplementary file 4 [file Table_2.docx]

Table S2. Species and NCBI accession numbers for the proteins used to construct a phylogenetic tree

| *Arabidopsis lyrata* | DXS1=XP_002870230.1 |
| --- | --- |
|  | DXS2=XP_002871472.1 |
|  | DXS3=XP_020886041.1 |
| *Antirrhinum majus* | DXS=AAW28999.1 |
| *Arabidopsis thaliana* | DXS1=NP_193291.1 |
|  | DXS2=NP_850620.2 |
|  | DXS3=NP_196699.1 |
| *Aegilops variabilis* | DXS=ABA19627.1 |
| *Brachypodium distachyon* | DXS1=XP_003568467.1 |
|  | DXS2=XP_003557443.1 |
| *Brassica rapa* | DXS1=XP_013737071.1 |
|  | DXS2=RID71922.1 |
|  | DXS3=RID42344.1 |
| *Chrysanthemum morifolium* | DXS=BAE79547.1 |
| *Carica papaya* | DXS1=XP_021904955.1 |
| *Catharanthus roseus* | DXS=CAA09804.2 |
| *Capsella rubella* | DXS1=XP_006283213.1 |
|  | DXS2=XP_023642413.1 |
| *Deinococcus radiodurans* | DXS=QEM70303.1 |
| *Escherichia coli* | DXS=EFF14228.1 |
| *Elaeis guineensis* | DXS1= NP_001290502.1 |
| *Ginkgo biloba* | DXS1=AAS89341.1 |
|  | DXS2=AAR95699.1 |
| *Hevea.brasiliensis* | DXS1=AAS94123.1 |
|  | DXS2=XP_021667494.1 |
| *Morinda citrifolia* | DXS=AAL32062.1 |
| *Morus notabilis* | DXS1=EXC03145.1 |
|  | DXS2A=XP_010102986.1 |
|  | DXS2B=XP_010113452.1 |
| *Mentha piperita* | DXS=AAC33513.1 |
| *Medicago truncatula* | DXS1=CAD22530.1 |
|  | DXS2=CAD22531.1 |
| *Narcissus pseudonarcissus* | DXS=CAC08458.1 |
| *Oryza sativa* | DXS1=XP_015640505.1 |
|  | DXS2=XP_015642490.1 |
|  | DXS3=XP_015647944.1 |
| *Picea abies* | DXS1=ABS50518.1 |
|  | DXS2=ABS50519.1 |
|  | DXS3=ABS50520.1 |
| *Pinus densiflora* | DXS1=ACC54557.1 |
|  | DXS2=ACC54554.1 |
| *Pueraria montana* | DXS=ACC54554.1 |
| *Physcomitrella patens* | DXS1=XP_024358572.1 |
|  | DXS2=XP_024380651.1 |
|  | DXS3=XP_024389704.1 |
|  | DXS4=XP_024379950.1 |
| *Populus trichocarpa* | DXS1=XP_006381844.1 |
|  | DXS2=XP_024460342.1 |
|  | DXS3=XP_006380580.2 |
|  | DXS4=XP_024463484.1 |
|  | DXS5=XP_024445465.1 |
| *Solanum habrochaites* | DXS=AAT97962.1 |
| *Setaria italic* | DXS1=XP_004962111.1 |
|  | DXS2=XP_004955719.1 |
|  | DXS3=XP_012701074.1 |
| *Solanum lycopersicum* | DXS1=AAD38941.1 |
| *Selaginella moellendorffii* | DXS1=XP_002965644.2 |
|  | DXS2=XP_002978764.2 |
| *Stevia rebaudiana* | DXS=CAD22155.2 |
| *Tagete erecta* | DXS=AAG10432.1 |
| *Taxus Media* | DXS=AAS89342.1 |
| *Zea mays* | DXS1=NP_001157805.1 |
|  | DXS2=NP_001295426.1 |
